# Anti-inflammatory effect of torilidis fructus ethanol extract through inhibition of Src

**DOI:** 10.1080/13880209.2017.1362011

**Published:** 2017-08-23

**Authors:** Gyubyung Park, Eunji Kim, Young-Jin Son, Deok Hyo Yoon, Gi-Ho Sung, Adithan Aravinthan, Yung Chul Park, Jong-Hoon Kim, Jae Youl Cho

**Affiliations:** a Gyeonggi Science High School for the Gifted, Suwon, Republic of Korea;; b Department of Genetic Engineering, Sungkyunkwan University, Suwon, Republic of Korea;; c Department of Pharmacy, Sunchon National University, Suncheon, Korea;; d Institute for Bio-Medical Convergence, International St. Mary's Hospital and College of Medicine, Catholic Kwandong University Incheon, Incheon, Republic of Korea;; e College of Veterinary Medicine, Chonbuk National University, Iksan, Republic of Korea;; f College of Forest and Environmental Sciences, Kangwon National University, Chuncheon, Republic of Korea

**Keywords:** Inflammatory mediators, NF-κB, nitric oxide, macrophages

## Abstract

**Context:** Torilidis fructus, fruits of *Torilis japonica* Decadolle (Umbelliferae), is a medicinal herb traditionally used as a pesticide, an astrictive, or a medicine for various inflammatory diseases.

**Objectives:** Due to the lack of pharmacological studies on this herbal medicine, we explored the inhibitory activity of torilidis fructus on the macrophage-mediated inflammatory response using its ethanol extract (Tf-EE).

**Material and methods:** The Griess assay and prostaglandin (PGE_2_) ELISA assay were conducted with Tf-EE (0-75 µg/mL) and LPS (1 µg/mL) treated RAW264.7 cells in cultured media. Tf-EE pretreated RAW264.7 cells were incubated with LPS for 6 h and semi-quantitative PCR was performed. Reporter gene assays, overexpression of target enzymes and immunoblotting were performed on macrophages to determine the molecular targets of Tf-EE.

**Results:** Tf-EE markedly suppressed the inflammatory response of macrophages, such as lipopolysaccharide (LPS)-induced nitric oxide (NO) and PGE_2_ production with IC_50_ values of 35.66 and 62.47 µg/mL, respectively. It was also found that Tf-EE reduced the expression of inducible NO synthase (iNOS) and cyclooxygenase (COX)-2 by 80%. Nuclear translocation and activation of nuclear factor (NF)-κB (p65 and p50) were declined by 60% and 30% respectively, and their regulatory events including the phosphorylation of AKT, IκBα, Src, and the formation of complexes between Src and p-p85 were also recognized to be diminished.

**Conclusions:** The signalling events managed by Src and p85 complex seemed to be critically involved in Tf-EE-mediated anti-inflammatory response. This might suggest that Tf-EE exhibited anti-inflammatory effects through Src-targeted inhibition of NF-κB.

## Introduction

Inflammatory responses protect the body from infection caused by bacteria, viruses, and fungi (Yu et al. 2012). To produce an inflammatory response, immune cells, such as macrophages, natural killer cells, and neutrophils, use special receptors such as toll-like receptors (TLRs) that recognize pathogen-derived material by its molecular pattern and that also regulate macrophage intracellular signaling (Chen et al. 2007). The activated receptors transduce several intracellular signaling cascades, such as those associated with Src, spleen tyrosine kinase (Syk), AKT (protein kinase B), inhibitor of IκB kinase (IKK), interleukin receptor-associated kinase (IRAK), mitogen-activated protein kinases (MAPK), and IκBα (Che et al. 2016; Park, Son, Kim, et al. 2016). This transduction activates certain transcription factors, nuclear factor (NF)-κB, and activator protein (AP)-1, which form transcription initiation complexes and trigger inflammatory gene expression, such as inducible nitric oxide synthase (iNOS), cyclooxygenase (COX)-2, and tumour necrosis factor (TNF)-α (Byeon et al. 2012; Baek et al. 2016; Tursun et al. 2016). This ultimately leads macrophages to produce toxic chemicals, such as nitric oxide (NO) and prostaglandin E_2_ (PGE_2_), and to phagocytize pathogens in order to decrease levels of harmful internal microbes (Page 1991). Inflammation will not be terminated if the infection condition that triggered the inflammatory response continues, which sometimes contributes to severe diseases such as cancer, diabetes, and Alzheimer's disease (Balkwill and Mantovani 2001; McGeer and McGeer 2001; Dandona et al. 2004). Therefore, there is a clinical need to suppress prolonged inflammation, and many studies are currently focusing on developing anti-inflammatory remedies.

*Torilis japonica* Decadolle (Umbelliferae) is an upright hedge parsley distributed throughout Japan, Korea, and China (Baskin and Baskin 1975). The fruit has traditionally been used as a pesticide, an astrictive, and a medicine for dermatopathy. Moreover, the anti-angiogenic activity, immune-enhancing activity, and antiviral activity of torilidis fructus have been reported (Kim et al. 2010). Although little correlative evidence between ethnopharmacological activity and this plant has been revealed, sesquiterpenoids like essential oils and hemiterpenoids have been identified as important ingredients (Kang et al. 1994; Ryu and Jeong 2001). As certain sesquiterpenoids from torilidis fructus have antibacterial activity toward several types of bacteria, some of these could be expected to be active components in its anti-inflammatory activity (Chen et al. 2013). Therefore, the anti-inflammatory effects of torilidis fructus were investigated. In addition, target molecules of the anti-inflammatory activity caused by Tf-EE, and potential active components were identified via investigation of its anti-inflammatory activity in macrophage-mediated inflammatory responses.

## Materials and methods

### Materials

3-(4,5-Dimethylthiazol-2-yl)-2,5-diphenyltetrazolium bromide (MTT), *N* ^ω^-nitro-l-arginine methyl ester hydrochloride (L-NAME), polyethylene imidazole (PEI), and lipopolysaccharide (LPS: *Escherichia coli* 0111:B4) were purchased from Sigma Chemical Co. (St. Louis, MO). PP2 was obtained from Callbiochem (La Jolla, CA). The enzyme immunoassay (EIA) kit for determining PGE_2_ levels was purchased from R&D systems (Minneapolis, MN). A luciferase construct containing the binding promoter for NF-κB was purchased from Promega (Madison, WI). Fetal bovine serum (FBS), DMEM, and RPMI 1640 were obtained from Gibco (Grand Island, NY). RAW264.7 and human embryonic kidney (HEK) 293 T cells were purchased from the American Type Culture Collection (Rockville, MD). All other chemicals were of Sigma grade. Antibodies against total protein and phosphoprotein for Flag, p65/NF-κB, p50/NF-κB, Akt, IκBα, p85/PI3K, Syk, Src, lamin A/C, and β-actin were obtained from Cell Signaling Technology (Beverly, MA). Antibody against HA protein was purchased from Santa Cruz (Santa Cruz, CA).

## Preparation of the ethanol extract (Tf-EE)

Tf-EE (code number: PBC-194AS) was obtained from the Plant Extract Bank in the Plant Diversity Research Center (Daejeon, Korea). Torilidis fructus was extracted using ethyl alcohol 95 & GR grade.

## Preparation of peritoneal macrophages

Male BALB/c, 7-week-old mice weighing 19–22 g, were obtained from Daehan Biolink (Eumseong, Korea) and maintained in plastic cages under conventional conditions. Water and a pelleted diet (Samyang, Daejeon, Korea) were supplied *ad libitum*. All animal studies followed the guidelines established by the Sungkyunkwan University Institutional Animal Care and Use Committee. Peritoneal exudates were obtained from 6-week-old male ICR mice via lavage for 4 d after 1 mL sterilized 4% thioglycollate broth (Difco Laboratories, Detroit, MI) was injected intraperitoneally (Park, Son, Kim, et al. 2016).

### Cell culture

RAW264.7 and HEK293T cells were cultured in RPMI1640 media and DMEM, respectively, supplemented with 10% heat-inactivated foetal bovine serum (Gibco, Grand Island, NY), glutamine, penicillin, and streptomycin at 37 °C with 5% CO_2_. For each experiment, the cells were detached with a cell scraper. When the cells were cultured for the experiments at a concentration of 2 × 10^6^ cells/mL, the % of dead cells was less than 1%, as assessed by trypan blue dye exclusion.

### NO production and Griess assay

RAW264.7 cells were plated at 1 × 10^6^ cells/mL onto a 96-well plate, incubated for 18 h, and pretreated with either Tf-EE (0-75 µg/mL) or other standard compounds (L-NAME, PP2, quercetin, kaempferol, and luteolin) for 30 min. Compound-treated cells were further incubated with LPS (1 µg/mL) for 24 h. The inhibitory effects of Tf-EE on LPS-induced NO production were determined by measuring NO levels using Griess reagent, as described previously (Cho et al. 2000; Kim et al. 2016).

### PGE_2_ production and ELISA assay

RAW264.7 cells (1 × 10^6^ cells/mL) were preincubated for 18 h, after which cells were treated with Tf-EE (0–75 µg/mL) for 30 min and then additionally treated with LPS (1 µg/mL) for 24 h. Using supernatant from RAW264.7 cells, the production of PGE_2_ was determined by ELISA kits according to the instructions from the manufacturer.

### Cell viability test

RAW264.7 cells were plated at 1 × 10^6^ cells/mL on a 96-well plate, incubated for 18 h and treated with Tf-EE (0–75 µg/mL) for 24 h. The cytotoxic effect of Tf-EE was evaluated using a conventional MTT assay, as reported previously (Pauwels et al. 1988; Park, Kang, et al. 2016). Briefly, 10 µL of MTT solution (10 mg/mL in phosphate-buffered saline, pH 7.4) was added to the cell culture media, and cells were incubated for 4 h and 15% sodium dodecyl sulphate was added to each well to solubilize the formazan. The absorbance at 570 nm (OD 570-630) of each well was measured using a SpectraMax 250 Microplate Reader (BioTek, Bad Friedrichshall, Germany).

### mRNA analysis by reverse transcription polymerase chain reaction

Total RNA was isolated from Tf-EE- and LPS-treated RAW264.7 cells with TRIzol Reagent (Gibco BRL, Carlsbad, CA) for the evaluation of inflammatory gene mRNA expression levels (Baek et al. 2015). The extracted RNA was stored at −70 °C until needed. The inhibitory effect of Tf-EE on mRNA expression levels was determined by reverse transcription polymerase chain reaction (RT-PCR), which was conducted as reported previously (Yang et al. 2015). Primers (Macrogen, Seoul, Korea) used are presented in Table 1.

**Table 1.** Primer sequences used in RT-PCR.

Name	Primer	Sequence (5′–3′)
iNOS	Forward	GGAGCCTTTAGACCTCAACAGA
	Reverse	TGAACGAGGAGGGTGGTG
COX-2	Forward	GGGAGTCTGGAACATTGTGAA
	Reverse	GCACATTGTAAGTAGGTGGACTGT
IL-1β	Forward	CAGGATGAGGACATGAGCACC
	Reverse	CTCTGCAGACTCAAACTCCAC
GAPDH	Forward	CAATGAATACGGCTACAGCACC
	Reverse	AGGGAGATGCTCAGTGTTGG

### Plasmid transfection and luciferase reporter assay

HEK293T cells (1 × 10^6^ cells/mL) were transfected with 1 µg of plasmid containing β-galactosidase (as a control) and NF-κB Luc in the presence or absence of an inducing molecule, Flag-MyD88, or CFP-TRIF. Transfections were performed using the PEI method in 6-well plates, as previously outlined (Hossen et al. 2016). Transfected cells were used at 48 h post-transfection for all experiments. Cells were treated with Tf-EE for the final 24 h of each experiment. Luciferase assays were performed using the Luciferase Assay System (Promega, Madison, WI), as previously reported (Sung et al. 2015).

### Preparation of cell lysate and nuclear fractions for immunoprecipitation and immunoblotting

RAW264.7 cells and HEK293T cells used to obtain cell lysates were washed in 1 mL cold phosphate-buffered saline (PBS) supplemented with 1 mM sodium orthovanadate three times, resuspended in lysis buffer (20 mM Tris-HCl, pH 7.4; 2 mM ethyleneglycotetraacetic acid [EDTA]; 2 mM ethyleneglycotetraacetic acid [EGTA]; 1 mM DTT; 50 mM β-glycerol phosphate; 0.1 mM sodium vanadate; 1.6 mM pervanadate; 1% Triton X-100; 10% glycerol; 10 µg/mL aprotinin; 10 µg/mL pepstatin; 1 mM benzamide; and 2 mM PMSF), and clarified by centrifugation at 12,000 rpm for 5 min at 4 °C and stored at −20 °C. RAW264.7 cells used for nuclear fractionation were treated in a similar way, but resuspended in nuclear protein extraction buffer A (10 mM HEPES with KOH, pH 7.8; 10 mM KCl; 2 mM MgCl_2_; 0.1 mM EDTA; 1 mM DTT; 0.1 mM PMSF; 2 µg/mL leupeptin; and 2 µg/mL aprotinin) and buffer B (10 mM HEPES with KOH, pH 7.8; 50 mM KCl; 400 mM NaCl; 0.1 mM EDTA; 1 mM DTT; 0.1 mM PMSF; 2 µg/mL leupeptin; 2 µg/mL aprotinin; and 10% glycerol) instead of lysis buffer. Soluble whole cell and nuclear lysates were immunoblotted, and total protein and phosphoprotein levels of p50, p65, IκBα, AKT, Syk, Src, p85/PI3K, HA, Flag, lamin A/C, and β-actin were determined, as previously reported (Park, Son, Aravinthan, et al. 2016).

### HPLC analysis

Constituents of Tf-EE were analyzed with high-performance liquid chromatography (HPLC) using an AZURA analytical HPLC system (KNAUER, Berlin, Germany). The HPLC system was equipped with a binary bump, an autosampler, and a water photodiode array detector. ClarityChrom software (Kanuer, Gaithersburg, MD) was employed for data acquisition. Tf-EE was analyzed using a Phenomenex column (Phenomenex, Torrance, CA) (Gemini C18, 5 µm, 250 × 4.60 mm). The HPLC mobile phases were H_2_O with 0.1% trifluoroacetic acid (v/v) as an aqueous mobile phase (A), and 95% MeCN with 0.08% trifluoroacetic acid (v/v) and 5% H_2_O as an organic mobile phase (B). The gradient conditions were as follows: 0–50 min, 100% A; and 50–60 min, 50% A. The flow rate was set at 1.0 mL/min. The HPLC chromatogram was recorded at 370 nm.

### Statistical analyses

All data are expressed as the mean ± SD of experiments performed with 6 samples. All other data presented are representative of three different experiments that yielded similar results. Similar experimental data were also obtained in an additional independent set of *in vitro* experiments that were performed with the same number of samples. For statistical comparisons, results were analyzed with an ANOVA combined with Scheffe’s *post hoc* test, and the Kruskal–Wallis and Mann–Whitney tests. A *p* value of < 0.05 was considered statistically significant. All statistical tests were performed with the SPSS software package (Version 22.0, 2013, IBM Corp., Armonk, NY).

## Results

### Effect of Tf-EE on NO and PGE_2_ production

To ascertain whether Tf-EE had an anti-inflammatory effect, NO levels from LPS-activated macrophages were preferentially determined. As seen in the left panel of Figure 1(A), Tf-EE suppressed production of NO in LPS-activated RAW264.7 cells, up to 97% at a dose of 75 µg/mL. NO levels in LPS-activated peritoneal macrophages were similarly suppressed by Tf-EE treatment in a dose-dependent manner, up to 90% at a dose of 75 µg/mL. Moreover, the dose-dependent NO inhibitory effect of L-NAME, a standard inhibitor known to block enzyme activity of NOS (Nicolarakis et al. 1994) in LPS-treated macrophages (Figure 1(A) right panel) indicated that the experimental environment was reliable, as previously reported (Jeong et al. 2014). Furthermore, we determined the level of another inflammatory mediator, PGE_2_, by ELISA assay (Figure 1(B)). Tf-EE clearly inhibited the production of PGE_2_. Since the observed NO inhibitory activity could be due to the cytotoxic effects of the compounds used, cell viability was determined using an MTT assay. As seen in Figure 1(C), there was no significant toxic effect on cell viability at up to a 75 µg/mL dose of Tf-EE or the standard compound L-NAME. Taken together, these data suggest that Tf-EE seemed to exert anti-inflammatory activity without affecting cell viability. To determine the component of Tf-EE that could explain its anti-inflammatory effects, HPLC was performed. As Figure 1(D) indicates, three major flavonoid components, quercetin, luteolin, and kaempferol, were detected at 35.3, 35.7, and 40.2 min after Tf-EE administration. The content of these compounds (quercetin, luteolin, and kaempferol) were revealed to be 0.003, 0.004, and 0.003%, respectively (Figure 1(D)). Finally, to confirm whether these compounds were able to suppress inflammatory responses, NO production levels were measured using LPS-treated RAW264.7 cells treated with these compounds. As we expected, these compounds significantly reduced the release of NO (Figure 1(E) left panel), without affecting cell viability (Figure 1(E) right panel).

Figure 1.IVF parameters as a function of serum DHEAS levels (A), androstenedione (B) and testosterone (C) in pregnant and non-pregnant women (linear regression).

**Figure:**
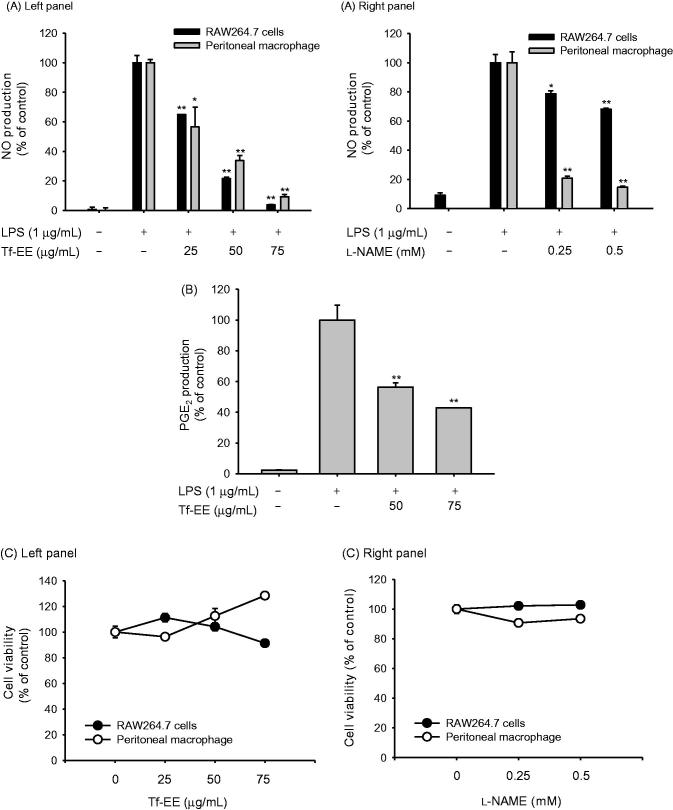

**Figure:**
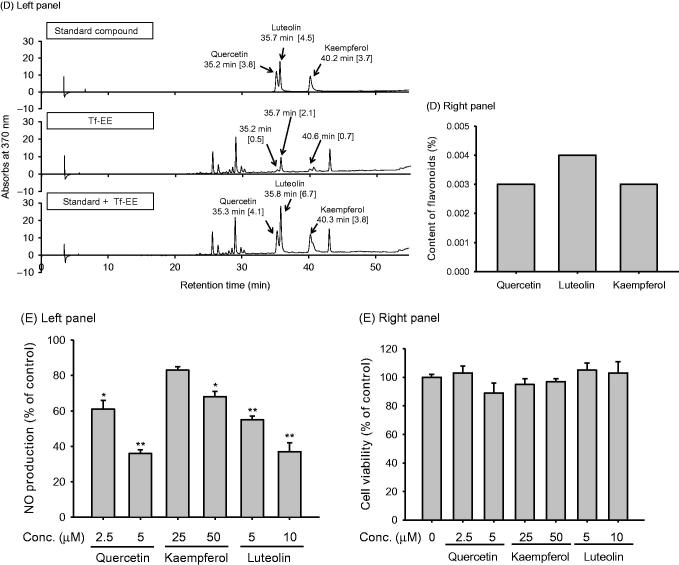

### Effect of Tf-EE on transcriptional activation of NF-κB

**Figure 2.**
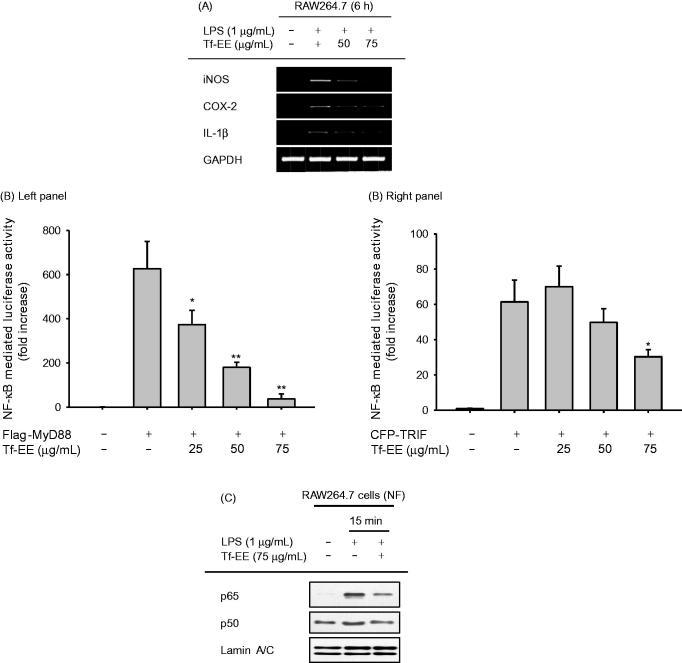
Effect of Tf-EE on transcriptional activation during TLR signaling. (A) mRNA levels of iNOS, COX-2, and IL-1β in RAW264.7 cells were measured by RT-PCR. (B) NF-κB activation was determined in NF-κB Luc and β-gal plasmid co-transfected HEK293T cells in the absence or presence of CFP-TRIF or Flag-MyD88. Luciferase activity was measured using a luminometer. (C) The levels of p65, p50, and lamin A/C in the nuclear fraction were determined by immunoblotting. NF: nuclear fraction. *p < 0.05 and **p < 0.01 compared with control.

To determine whether the inhibition of NO production caused by Tf-EE was regulated at the transcriptional level, mRNA levels were first assessed by RT-PCR. As seen in Figure 2(A), LPS strongly increased iNOS, COX-2 and IL-1β at the mRNA level in RAW264.7 cells. Of note, iNOS and COX-2 mRNA levels were suppressed by treatment with Tf-EE (50–75 µg/mL), and IL-1β expression was slightly decreased by Tf-EE. This demonstrated that Tf-EE affected transcription of these genes. It has been previously reported that expression of iNOS and COX-2 was promoted by NF-κB (Tak and Firestein 2001). As NF-κB plays important roles in modulating the expression of inflammatory genes, the potential suppression of activation and translocation of NF-κB by Tf-EE was examined by a reporter gene assay and immunoblotting. A reporter gene assay using HEK293T cells treated with Flag-MyD88 or CFP-TRIF was chosen to check the suppression of NF-κB activation. As seen in Figure 2(B), treatment with Flag-MyD88 and CFP-TRIF increased luciferase activity, while Tf-EE dose dependently suppressed said activity in HEK293T cells. To confirm that the suppression of NF-κB activity by Tf-EE also led to the blocking of NF-κB translocation, levels of nuclear p65 and p50, which compose NF-κB, were determined by nuclear fractionation and immunoblotting analysis from lysates obtained 15 min after treatment with LPS in the presence of Tf-EE. Tf-EE strongly inhibited nuclear p65 in RAW264.7 cells as expected; suppression of nuclear p50 was also observed (Figure 2(C)).

### Effect of Tf-EE on the NF-κB activation pathway

**Figure 3.**
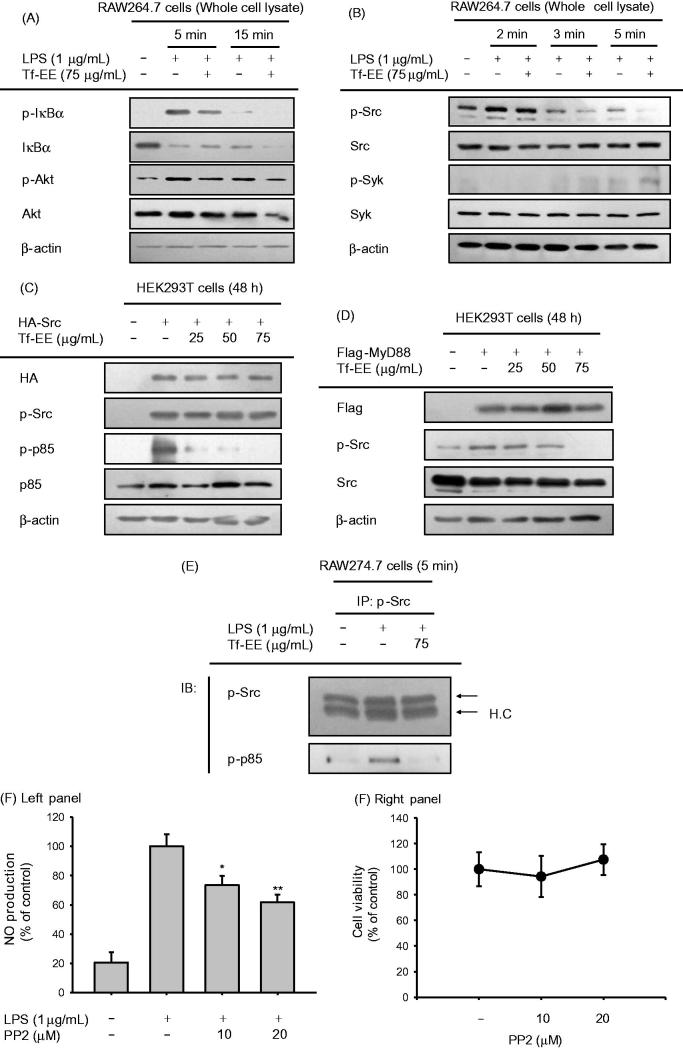
Effect of Tf-EE on the NF-κB activation signaling cascade. (A and B) The levels of total protein and phosphoprotein of IκBα, AKT, Syk, Src and β-actin in the whole cell lysates were determined by immunoblotting. (C) HA-Src plasmid transfected HEK293T cells were treated with Tf-EE. The levels of p-Src, HA, p-p85, p85 and β-actin in whole cell lysates were determined by immunoblotting. (D) Flag-MyD88 plasmid-transfected HEK293T cells were treated with Tf-EE. The levels of Flag, p-Src, Src and β-actin in whole cell lysates were determined by immunoblotting. (E) The levels of molecular complexes between Src and p-p85 were measured in LPS-induced RAW274.7 cells with Tf-EE treatment by immunoblotting after immunoprecipitation. (F) A Src inhibitor, PP2, was pretreated on the LPS-treated RAW264.7cells. NO levels were measured via Griess assay. *p< 0.05 and **p< 0.01 compared with control.

Since activation and translocation of NF-κB were suppressed by treatment with Tf-EE, the pharmacological target of Tf-EE with respect to its anti-inflammatory activity could be the NF-κB activation pathway. Therefore, total protein and phosphoprotein levels of IκBα and AKT, which are part of the upstream signalling cascade for activation of NF-κB, were determined by immunoblotting. As seen in Figure 3(A), Tf-EE clearly suppressed phosphorylation of IκBα and AKT at 5 and 15 min after treatment with Tf-EE. As phosphorylation of Syk and Src is known to increase phosphorylation of IκBα and AKT (Lee et al. 2009; Byeon et al. 2012), we investigated the levels of p-Syk and p-Src by immunoblotting at 2, 3, and 5 min after treatment with Tf-EE. We found that Tf-EE only suppressed levels of p-Src at 3 and 5 min after LPS treatment (Figure 3(B)).

Src plays a pivotal role in inflammatory responses (Byeon et al. 2012), and our data support that it may be a pharmacological target protein of Tf-EE. Therefore, we assessed whether Tf-EE directly inhibited the phosphorylation of p85, which is affected by the phosphorylation of Src and which also activates the NF-κB pathway, by immunoblotting cell lysates derived from HEK293T cells transfected with Src plasmid DNA. As seen in Figure 3(C), the upregulated levels of p-p85 by Src overexpression were strongly suppressed by treatment with Tf-EE (concentration range: 25–75 µg/mL). Since the level of p-Src was not affected by Tf-EE (Figure 3(C)), further investigation determined the levels of p-Src under the presence of Flag-MyD88, which directly stimulates activation of Src, in transfected HEK293T cells to confirm that Tf-EE targeted Src as part of its anti-inflammatory response. Our results showed that the phosphorylation of Src was inhibited by Tf-EE in transfected HEK293T cells under the presence of Flag-MyD88 (Figure 3(D)). Furthermore, to confirm that Tf-EE inhibited the activation of Src, the formation of molecular complexes between Src and the active form of its substrate protein (p-p85) with regulatory function for the activation of PI3K (Walker et al. 2007) was determined by immunoprecipitation followed by immunoblotting. As seen in Figure 3(E), p-p85 and p-Src complex formation was suppressed by Tf-EE in RAW274.7 cells. Finally, NO production from LPS-activated RAW264.7 cells was strongly inhibited by a specific inhibitor of Src, PP2, confirming that Src played an important role in inflammatory responses (Figure 3(F)).

## Discussion

In this study, the anti-inflammatory activity of torilidis fructus, which is distinct from its traditional and more well known uses as a pesticide, astrictive and in the treatment of dermatopathy, was tested at the cellular level in LPS-activated macrophages. LPS interacts with TLRs and induces macrophages to produce inflammatory mediators like NO and PGE_2_ (Chen et al. 2007), which maintain the defensive function and determine blood flow rate (Cals-Grierson and Ormerod 2004). Tf-EE effectively suppressed upregulation of NO and PGE_2_ levels (Figure 1), implying that Tf-EE can inhibit the inflammatory response of LPS-activated macrophages. Importantly, not only iNOS and COX-2 were reduced at the mRNA level by Tf-EE treatment under the same conditions (Figure 2(A)) but also suppression of the NF-κB pathway was found (Figure 2). Considering that NF-κB plays a wide variety of important roles, such as the development and differentiation of dendritic cells (Tak and Firestein 2001), and also given that many NF-κB inhibitors, such as resveratrol and luteolin, have been developed as anti-inflammatory remedies (Kwak et al. 2011; Panaro et al. 2012; Lee et al. 2015), it is likely that Tf-EE could be used as an anti-NF-κB inhibitory remedy with similar effects.

To understand the anti-inflammatory activity of Tf-EE at a molecular level, further studies focused on distinguishing the pharmacological target in the NF-κB pathway of Tf-EE were performed. Activation of NF-κB (Figure 2(B)), translocation of p65, which is a key component of NF-κB, at 15 min (Figure 2(C)), and phosphorylation of both AKT and IκBα were inhibited by Tf-EE in RAW264.7 and HEK293T cells (Figure 3(A)). Considering the fact that it has been reported that phosphorylation of IκBα at 5 min during LPS stimulation is regulated by the tyrosine kinases Syk and Src (Lee et al. 2009), it was expected that Tf-EE could inhibit phosphorylation of Syk or Src as part of its anti-inflammatory activity. While no significant change in the levels of p-Syk was observed, treatment with Tf-EE suppressed the level of p-Src at 3 and 5 min after treatment, as expected (Figure 3(B)). Moreover, events downstream of Src, such as the phosphorylation of p85 in Src-overexpressing HEK293 cells (Figure 3(C)) and the formation of a molecular complex between Src and p-p85 in LPS-activated macrophages (Figure 3(E)), were definitively inhibited by Tf-EE treatment. Since Src has already been shown to be an important signalling molecule in a variety of immunological processes, including macrophage-mediated inflammatory responses (Byeon et al. 2012), Tf-EE may directly target Src as its anti-inflammatory activity. In addition, inhibitors of Src have been already reported to suppress the production of proinflammatory cytokines, such as TNF-α and IL-1β (Byeon et al. 2012). Therefore, our results strongly suggest that Tf-EE directly targets Src as part of its anti-inflammatory process.

Since Tf-EE seemed to have anti-inflammatory activity on macrophages, it was important to determine the active components of Tf-EE. Until now, few reports have shown that some essential oils and sesquiterpenoids can be separated from torilidis fructus (Hwang et al. 2007). Since no component of this plant has been previously shown to have anti-inflammatory activity, HPLC was done to determine the flavonoids present in Tf-EE. As Figure 1(C) shows, flavonoids that we expected to be in the Tf-EE were found (quercetin, kaempferol, and luteolin). Since these compounds were reported to suppress the phosphorylation of Src and its kinase activity (Endale et al. 2013; Hossen et al. 2015; Kim et al. 2015; Lee et al. 2015), effects of Tf-EE on the phosphorylation of Src (Figure 3) as well as NO production (Figure 1) might be contributed to by these compounds. In particular, luteolin was recently found to directly bind to the ATP-binding domain SH1 of Src (Lee et al. 2015). This finding seems to open the possibility that some Tf-EE flavonoids might suppress Src enzyme activity by competing with ATP in the Src kinase domain. To show this, flavonoid-binding amino acids need to be identified by using mutant constructs of Src in ATP binding domains by employing point mutations. Nonetheless, since there are many peaks between 25 to 30 min, future research will be focused on identifying other Tf-EE compounds showing anti-inflammatory activities as well as flavonoid-binding amino acids.

**Figure 4.**
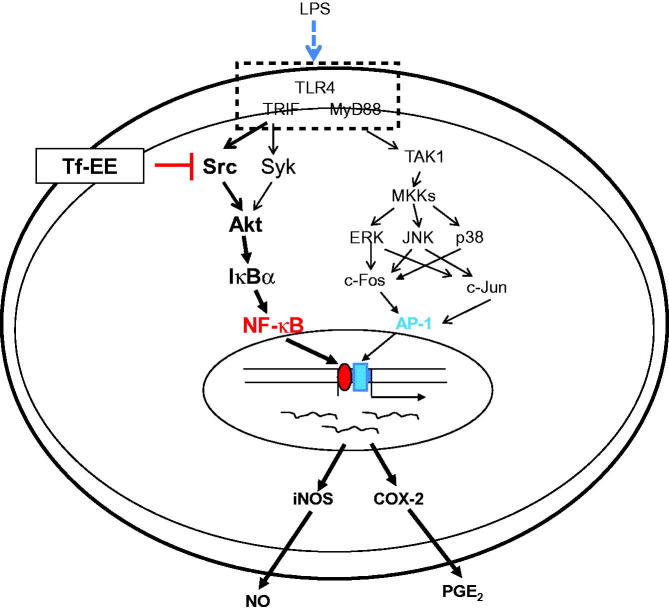
Putative inhibitory pathway of LPS-activated inflammatory responses mediated by Tf-EE.

In summary, our study suggests a new function for Tf-EE in the suppression of NO at the transcriptional level in LPS-activated macrophages. Moreover, we demonstrated that this anti-inflammatory activity was triggered by inhibition of the Src/NF-κB pathway, as summarized in Figure 4. By HPLC, it was found that quercetin, kaempferol, and luteolin, known to suppress the phosphorylation of Src, were included in Tf-EE. Therefore, further studies should be done to determine other active components of Tf-EE, and to further investigate its anti-inflammatory effects using an *in vivo* model, in order to confirm its possible utility as an anti-inflammatory remedy.
